# A Modified Through and Through Guidewire Steerable Sheath Technique for Transfemoral Access to Visceral Arteries with Hostile Anatomy

**DOI:** 10.1016/j.ejvsvf.2021.05.001

**Published:** 2021-05-18

**Authors:** Marcell Gyánó, Csaba Csobay-Novák

**Affiliations:** a Weba Heart and Vascular Center, Semmelweis University, Budapest, Hungary; b Department of Interventional Radiology, Semmelweis University, Budapest, Hungary

**Keywords:** Hostile anatomy, Steerable catheter, Visceral stenosis, Endovascular technique

## Introduction

A 69 year old woman was admitted with chronic mesenteric ischaemia. Her medical history included left subclavian transposition followed by a transthoracic endovascular aortic repair between zones 2 and 5, and a complete relining of the endografts years later owing to a type V endoleak. To avoid crossing the hostile arch and the descending aorta, and to achieve a stable position for cannulation of the superior mesenteric artery (SMA) with a steep take off, a steerable catheter was prepared from conventional devices based on the idea of the through and through suture technique.^1^

## Surgical technique

**Figure 1:**
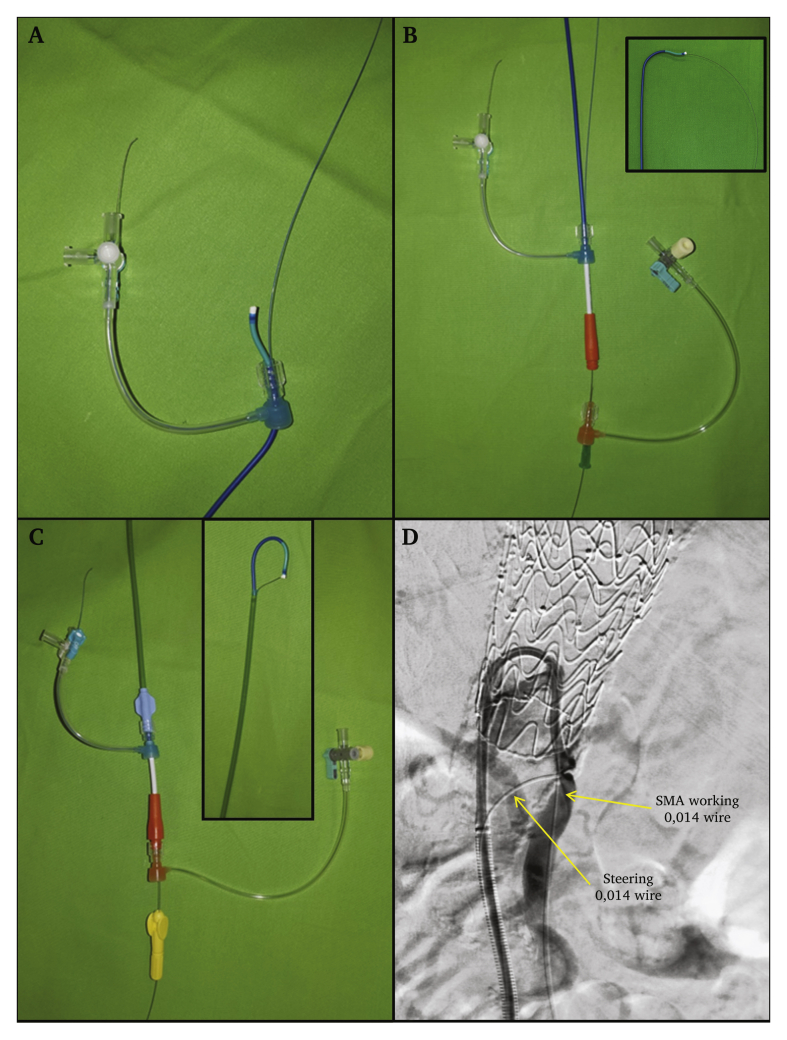
(A) Eight French side port retrograde cannulation with a 0.014 guidewire and the 7 F AL2 catheter pushed through the 8 F valve (near the 0.014 wire). (B) The 0.014 wire was pulled through an off centre needle puncture of the 7 F valve, without narrowing the working diameter of the valve. (Inset) Tip of the AL2 catheter. (C) The assembled device with steered back 7 F AL2 catheter. (D) The device in use.

An ultrasound guided right common femoral artery puncture was used. A 65 cm long 8 F sheath (Destination; Terumo, Tokyo, Japan) was advanced in the aorta up to the level of the visceral arteries. The valve and the side port were then removed and replaced with a cap for the time of the preparation. It is essential to use a sheath with a removable valve to use this technique. The 8 F side port was cannulated in a retrograde fashion with the soft tip of a 300 cm 0.014 guidewire and was secured with the side port pin. A 7 F guide catheter (Launcher AL2; Medtronic, Minneapolis, MN, USA) was pushed through the 8 F valve. The back end of the wire was then inserted into the 7 F catheter in a retrograde fashion. At the hub of the 7 F catheter a separate haemostatic valve was used (Radifocus Hemostasis Valve; Terumo). The wire was pulled through an off centre needle puncture of the 7 F valve and was bent in half at the tip of the guide catheter. The 7 F guide catheter was then inserted into the sheath with the 8 F valve positioned near the hub of the 7 F guide catheter, advanced completely, and, finally, the 8 F valve was reconnected to the sheath. The 7 F catheter could be steered with a pull on the steering wire (see Fig. 1).

Once the steerable sheath was in place and oriented against the SMA ostium, the stenosis was crossed with a standard 0.014 guidewire. As the bowstringed guide catheter provided enough support, direct stenting was performed using a 6 × 12 mm balloon expandable stent (Dynamic Renal; Biotronik, Bülach, Switzerland). The access point was closed using an 8 F closure device (Angio-Seal VIP; Terumo). The patient was ambulant two hours later and discharged the following day.

## Discussion

Renovisceral arteries with a steep take off associated with a hostile arch or descending aorta (e.g., elongated or shaggy) can be handled more safely via femoral access, without the need for crossing the arch. This can be done using either a dedicated small calibre steerable sheath (6–8.5 F), which usually lacks strong antegrade support, or a large steerable sheath (16 F), which has better support but adds a non-negligible bleeding risk given the large bore arterial access.

The advantage of the described technique is that it provides the best of both worlds: an antegrade approach for the caudally oriented visceral branches with the ease of more direct control using shorter devices from transfemoral access. The bowstringed guide catheter provides a strong support even for those occlusions and tight stenoses where dedicated steerable sheaths tend to lose support and straighten during lesion crossing. This is clearly the Achilles heel of every steerable sheath currently available and the reason why homemade steerable devices are gaining popularity in transfemoral access to branched endovascular aortic repair.

An additional advantage is that this setup can be produced from standard devices that are usually available in every catheterisation laboratory. It can be also useful in urgent settings when a dedicated steerable sheath is not readily available. Last, but not least, the devices used for this technique costs much less than the cheapest dedicated steerable sheath.

### Conclusion

This cost effective, homemade setup provided a strong support for cannulation of a visceral artery with a hostile take off and to cross the ostial stenosis easily with all the devices needed. This setup can be use also in the treatment of primary atherosclerotic lesions and in cases with complications.

## Funding

None.
